# Closed-Loop Extracorporeal Vascular Cleaning by Staged Chemical Dissolution

**DOI:** 10.7759/cureus.108455

**Published:** 2026-05-07

**Authors:** Ilia Toli

**Affiliations:** 1 Materials Science and Engineering, University of Texas at Dallas, Richardson, USA

**Keywords:** atherectomy, atherosclerosis, bifurcation lesions, chemical dissolution, collagenase, edta chelation, extracorporeal circulation, interventional cardiology, plaque regression, vascular calcification

## Abstract

A closed-loop extracorporeal system for the removal of atherosclerotic plaque from accessible vascular segments by targeted chemical dissolution in a blood-free environment is proposed. The target segment is isolated by balloon occlusion, drained of blood, and connected to an external bypass that maintains distal perfusion. With the treatment zone converted to a sealed saline chamber, concentrated chemical agents, each targeting a specific plaque component, dissolve the atheroma in situ: ethylenediaminetetraacetic acid (EDTA) for calcified deposits, surfactants for the lipid-rich necrotic core, and collagenase for the fibrous cap. The agents are cycled iteratively to penetrate layered plaque, and dissolved material is flushed out under alternating unidirectional flow, with real-time effluent chemistry providing both treatment endpoint and quantitative plaque composition without imaging. The access geometry uses n+1 balloon catheters to isolate arbitrary vessel topologies, from straight segments to bifurcations and higher-order branch points. An iterative protocol interleaves gentle cleaning sessions with pharmacological lipid and calcium mobilization, progressively draining plaque burden from inaccessible microvascular sites toward accessible ones. Six testable predictions are specified for validation in a porcine atherosclerosis model.

## Introduction

Every existing percutaneous atherectomy device, namely, rotational (Rotablator, Boston Scientific, Marlborough, Massachusetts, United States), orbital (Diamondback 360, Cardiovascular Systems Inc., Saint Paul, Minnesota, United States), excimer laser (CVX-300 ELCA, Philips/Spectranetics, Colorado Springs, Colorado, United States), and directional (SilverHawk/TurboHawk, Medtronic, Minneapolis, Minnesota, United States), operates within a systemically connected lumen [1]. These devices, developed across four decades since the introduction of rotational atherectomy in the late 1980s, share a common architectural premise: plaque is disrupted in flowing blood, and debris generated during disruption enters the bloodstream. Distal embolization protection devices capture only a fraction of particulate [2]. This forces a trade-off that has governed the field for four decades: aggressive enough to remove plaque and gentle enough that escaped debris does not cause end-organ damage.

A second constraint is equally binding: no chemical agent can be used at therapeutic concentration against plaque in a systemically connected vessel. Ethylenediaminetetraacetic acid (EDTA) at the concentration needed to dissolve calcified plaque (50-100 mM, approximately 10-50 times higher than systemic infusion) would chelate the ionized calcium required for coagulation. Collagenase at the concentration needed to digest a fibrous cap (typically 0.1-0.5 mg/mL of bacterial collagenase) would attack every collagen-containing structure in the body. Surfactants at the concentration needed to emulsify a lipid core (poloxamer 188 at 1-5% w/v or hydroxypropyl-β-cyclodextrin at 10-50 mM) would lyse red blood cells or perturb membrane integrity systemically. The chemistry that would dissolve atheroma is well characterized: it simply cannot be deployed in blood.

Both constraints follow from a single premise: that the treatment zone is part of systemic circulation. Remove that premise, and both constraints vanish simultaneously.

## Technical report

Core principle: topological isolation of the treatment zone

**Figure 1:**
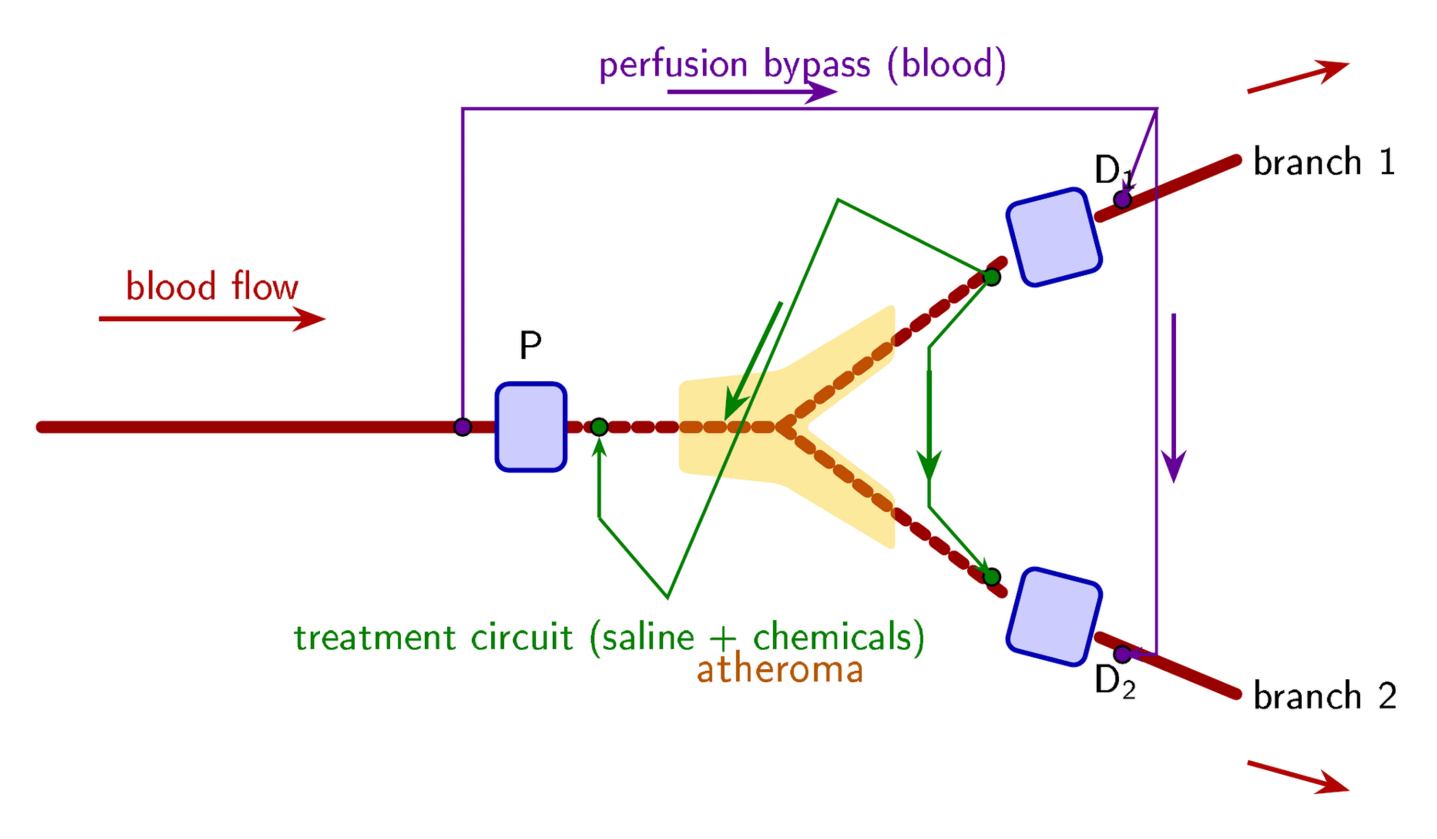
Access geometry of the closed-loop extracorporeal vascular cleaning system Each balloon catheter is a dual-lumen device with ports on opposite sides of its balloon. Blood-side ports (purple) connect to the perfusion bypass; chamber-side ports (green) connect to the treatment circuit. In this bifurcation example, three balloons (P, D1, D2) isolate the entire branch point. A single external bypass with Y-split maintains perfusion to both daughter branches. Dashed lines indicate the saline-filled isolated chamber where chemical dissolution occurs. Three sets of flow direction arrows are shown: red arrows indicate native blood flow (parent vessel inflow, daughter branch outflow); purple arrows indicate the perfusion bypass circuit direction; green arrows indicate the treatment circuit direction. The system generalizes to arbitrary vessel topology with n+1 balloons for n downstream branches. Figure created with TikZ/PGF (LaTeX).

Two balloon catheters are placed percutaneously by the Seldinger technique [3], bracketing the target lesion under ultrasound guidance. Each catheter is a dual-lumen device with ports on opposite sides of its balloon. Balloon inflation seals the vascular segment; the ports on each side of each balloon connect the catheter to the two independent circuits that the system requires (Figure 1).

The bypass circuit carries blood. The proximal catheter's upstream port, opening into flowing blood proximal to the balloon, taps arterial blood into an external heparin-coated bypass line. This line returns blood through the downstream ports of the distal catheters, opening into the daughter branches beyond the distal balloons. The bypass is functionally identical to the shunt circuits used in carotid endarterectomy and cardiopulmonary bypass [4]. Systemic anticoagulation is achieved with unfractionated heparin: a loading dose of 70-100 IU/kg administered intravenously prior to bypass initiation, with maintenance dosing titrated to an activated clotting time (ACT) target of 250-300 s, monitored at 30-minute intervals throughout the procedure. This is the standard heparinization protocol for percutaneous coronary intervention with mechanical circulatory support [5]. The patient experiences no ischemia.

The treatment circuit carries saline and chemicals. The proximal catheter's downstream port, opening into the sealed chamber distal to the balloon, injects chemical agents into the isolated segment. The distal catheters' upstream ports, opening into the chamber proximal to the distal balloons, extract effluent. The treatment circuit is a closed saline loop: chemical reservoirs, proximal catheter, isolated vessel segment, distal catheters, effluent monitoring and filtration, and return to reservoirs. The pump is bidirectional.

After balloon inflation and bypass establishment, residual blood in the isolated segment is flushed out through the treatment circuit until the segment is blood-free. What remains is a sealed saline chamber, topologically disconnected from systemic circulation. Within this chamber, debris size and composition are irrelevant because nothing enters systemic circulation; chemical agents can be used at any concentration because no blood cells and no coagulation cascade are present; treatment duration is unconstrained by biological clocks because no stagnant blood activates platelets or complement; effluent monitoring operates against a clean saline baseline instead of the noise floor of whole blood.

The bypass circuit carries blood exclusively. The treatment circuit carries saline and chemical agents exclusively. The two circuits share the same catheters but are separated by the balloon seals. They never mix during the procedure.

Minor branch occlusion

No clinically relevant arterial segment is free of minor branches. Intercostal arteries, genicular branches, and the superior thyroid artery are examples of small branches that arise every few centimeters throughout the major vessels. Balloon isolation of any segment necessarily occludes these branches temporarily, depriving their perfusion territories of blood flow for the duration of treatment.

This is clinically routine. Every balloon angioplasty occludes side branches for the duration of inflation, and interventional cardiologists manage this daily with decades of safety data. Treatment duration per segment (tens of minutes to one hour) is within the established safety window.

Chemical agents in the saline chamber will enter minor branch ostia. This is partially therapeutic: branch ostia are themselves atheroprone sites due to disturbed flow at the branch point. EDTA dissolving calcium at a branch ostium, or surfactant clearing lipid from a small branch origin, provides incidental cleaning of sites that could not be accessed intentionally. However, EDTA at 50-100 mM entering a small branch could chelate intracellular calcium from smooth muscle cells, potentially affecting vascular tone. The concentration reaching deeper branch tissue will be attenuated by diffusion and dilution as a function of branch diameter and treatment circuit flow rate, but the safe exposure envelope for minor branches requires empirical characterization in the animal model. Everything is flushed completely before blood is restored.

Balloon seal integrity

The entire architecture depends on fluidic isolation at the balloon-vessel interface. Balloon seals can leak, particularly in calcified, eccentric vessels, precisely the vessels this system targets. Leakage of EDTA or collagenase past a balloon into flowing blood would be dangerous.

The primary safeguard is a pressure differential regime: the treatment circuit is maintained at pressure slightly below the bypass circuit at all times. Under this condition, any leakage at the balloon seal is inward (blood leaking into the chamber), not outward (chemical agents escaping into the blood). Inward leakage dilutes the treatment agents but poses no systemic risk; the effluent monitoring system detects it as a rise in hemoglobin absorbance in the saline stream.

Continuous pressure differential monitoring between the two circuits provides real-time seal integrity confirmation. If treatment circuit pressure approaches or exceeds bypass pressure, indicating a seal compromise that could allow outward leakage, the abort protocol is immediate: treatment circuit flushed to saline, balloons deflated, and procedure terminated. Sentinel chemistry (ionized calcium assay on bypass circuit blood sampled at the distal catheter) provides a secondary leak detection channel specific to EDTA escape.

Modern compliant balloons conform to irregular vessel geometry better than early non-compliant designs, but heavily calcified vessels with eccentric lumens remain the most challenging seal environment. Vessel segments with severe circumferential calcification may require pre-treatment assessment of seal adequacy (test inflation with saline before introducing chemical agents) and may, in some cases, be unsuitable for this protocol, requiring surgical endarterectomy instead.

Predilection sites and access geometry

Atherosclerotic plaque does not distribute uniformly. It accumulates preferentially at sites of disturbed hemodynamic shear stress: low and oscillatory wall shear stress zones [6]. These sites are predominantly branch points (bifurcations, trifurcations, ostial takeoffs), arterial curvatures, and the proximal faces of pre-existing stenoses. Branch lesions are also among the most difficult targets for conventional percutaneous intervention, because stenting or atherectomy of the main vessel frequently compromises the side branch [7].

The access geometry generalizes to arbitrary vessel topology. An uncomplicated straight segment requires two balloon catheters, one at each end. A bifurcation requires three (one proximal, one in each daughter branch). A trifurcation or higher-order branch point requires n+1 catheters for n downstream vessels, with a proportional increase in the bypass manifold. The perfusion bypass line from the proximal catheter's blood-side port splits through a Y- or X-connector to return blood through each distal catheter's blood-side port, maintaining perfusion to all downstream territories. Chemical agents in the treatment circuit access every surface of the isolated region from all directions.

The isolated region is a volume, not a single vessel. Any branch arising within the balloon-bracketed segment is part of the treatment chamber and is cleaned simultaneously with the main segment. A single treatment session can therefore address a primary vessel together with the secondary branches that take off within it, which matches the clinical reality that disease at a branch point affects the main vessel and the ostia of its daughters as a single unit.

The system is not restricted to bifurcations; it is agnostic to the branch count of the isolated segment. Bifurcations are the principal clinical target because that is where the disease concentrates. Principal treatment sites include the coronary arteries (femoral or radial access), the carotid bifurcation (direct cervical access), the aortic bifurcation into the common iliacs (bilateral femoral access), the femoral bifurcation (direct inguinal access), the renal artery ostia (femoral access), the aortic arch branches (femoral plus upper extremity access), and the subclavian and vertebral vessels (upper extremity access).

Bidirectional flow

With blood removed from the treatment zone, flow directionality is a free parameter. The pump operates in sustained directional passes, full forward transit and then full reverse transit, not oscillation.

Plaque geometry is three-dimensional: overhangs, shoulders, and recesses behind calcified nodules. Unidirectional flow creates chemical shadow zones on the downstream face of every surface feature. Directional reversal ensures every surface is alternately upstream and downstream, receiving fresh chemical agent from both sides. In a branched geometry (Figure 1), flow can be directed from the parent vessel into each branch alternately and reversed from each branch back through the parent, ensuring that branch ostia, carinae, and flow dividers, where the densest plaque typically sits, are attacked from every angle.

Each directional pass produces an independent effluent signal. If the forward pass through one branch shows elevated calcium but the reverse pass runs clean, the calcification is localized to the proximal face of that branch. Per-direction effluent analysis provides spatial information about plaque distribution without imaging. The treatment endpoint is bilateral convergence: effluent from all directions simultaneously at baseline. This is a strictly stronger completion criterion than unidirectional convergence.

Staged chemical dissolution

Each major component of the atheroma has a specific, well-characterized chemical vulnerability. The protocol exploits these in repeated cycles, with a saline rinse between agents to prevent inter-agent reactions. Because atheromata are layered, calcium entombed in lipid wrapped in fibrous cap, with more calcium beneath, a single pass of any agent cannot reach the full depth of its target. The stages are cycled: EDTA, rinse, surfactant, rinse, collagenase, rinse, then EDTA again, and so on, until all effluent signals converge to baseline simultaneously. Each cycle penetrates deeper because the preceding agents have removed the overlying material.

The stages are synergistic. EDTA dissolving calcium destabilizes the matrix that holds lipid in place, making the subsequent surfactant pass more effective. Surfactant removing lipid exposes buried calcified nodules to the next EDTA pass. Collagenase digesting fibrous cap exposes the lipid core beneath. Each agent, acting on its specific target, incidentally loosens every other component. The total effect exceeds the sum of the individual stages.

The mechanism of progressive penetration is substrate cohesion. Plaque integrity is determined less by each layer's intrinsic mechanical properties than by each layer's bond to its substrate. A tough fibrous cap can be removed once the lipid core beneath has lost cohesion through surfactant action; a moderately calcified deposit can be flushed out once the matrix it was embedded in has been chemically softened, even though the calcium itself remains hard. The 1,000-fold range of mechanical properties within a single plaque (from soft lipid core to hard calcified deposits) becomes manageable because chemistry attacks substrates rather than requiring mechanical disruption of each component at its native hardness. This is consistent with how plaque rupture occurs clinically, a failure of the cap-core interface rather than of the cap's intrinsic strength, applied here as a controlled removal mechanism rather than a pathological event.

Stage 1: Calcium Dissolution

Disodium EDTA in sterile saline at 50-100 mM is used to chelate Ca²⁺ directly from the hydroxyapatite crystal lattice, the hardest plaque component. Hydroxyapatite has a Vickers hardness of approximately 60 HV (HV quantifies indentation resistance on a scale ranging from approximately 5 HV for lead to approximately 10,000 HV for diamond). The chelation reaction dissolves the calcium into soluble calcium-EDTA complexes. EDTA chelation has been tested intravenously at systemic concentrations in the Trial to Assess Chelation Therapy [8], which demonstrated cardiovascular benefit in diabetic post-myocardial infarction patients despite the low concentration required for systemic safety. In the present system, with no coagulation cascade to disrupt, EDTA can be used at concentrations 10-50 times higher than systemic infusion, dissolving calcified deposits that no pharmacological agent and no existing interventional device can regress. The effluent signal for this stage is calcium concentration measured by ion-selective electrode. Published dissolution rates for hydroxyapatite in EDTA at neutral pH range from 10 to 50 micrograms per square centimeter per minute depending on crystal morphology and stirring conditions [9]. A calcified nodule with 50 mg of calcium and 1 cm² exposed surface would require several hours of continuous EDTA exposure at these rates, longer than a practical single-session treatment window. However, three factors accelerate the effective rate: the iterative cycling protocol continuously exposes fresh calcium surface as overlying lipid and fibrous material are removed by alternating agents; calcification in atheroma is distributed through the plaque matrix rather than consolidated in a single block, presenting a much larger effective surface area than a compact nodule; and the bidirectional flow regime prevents diffusion-limited stagnation zones. The actual dissolution rate within a layered atheroma under cycling conditions is an empirical question that the porcine atherosclerosis model experiments are designed to answer.

Stage 2: Lipid Emulsification

Poloxamer 188 at 1-5% weight by volume in saline, or hydroxypropyl-β-cyclodextrin at 10-50 mM, is used as the surfactant agent.** **Poloxamer 188 is a nonionic surfactant that emulsifies the cholesterol-rich necrotic core into a flushable emulsion. Hydroxypropyl-β-cyclodextrin solubilizes cholesterol directly by inclusion-complex formation and has been shown to promote atherosclerosis regression in animal models [10]. Poloxamer 188 has been used intravenously in clinical trials for sickle cell vaso-occlusive crisis [11]. Hydroxypropyl-β-cyclodextrin is the solubilizing excipient in injectable drug formulations including voriconazole. Both have established intravenous safety profiles; in the isolated saline compartment, they can be used at concentrations far exceeding systemic limits. The effluent signals for this stage are turbidity, measured by nephelometry, and lipid concentration, measured by enzymatic colorimetry.

Stage 3: Fibrous Cap Digestion

Collagenase from *Clostridium histolyticum* at 0.1-0.5 mg/mL in buffered saline is used to digest the fibrous cap. This is the same enzyme used in the US Food and Drug Administration (FDA)-approved Xiaflex for Dupuytren's contracture [12]. The enzyme cleaves collagen types I and III at specific glycyl-isoleucyl and glycyl-leucyl bonds, digesting the fibrous cap into soluble peptide fragments. The vessel media also contains collagen, but is shielded beneath the internal elastic lamina, which is composed of elastin, a substrate collagenase does not cleave. In segments where the lamina is intact, it functions as a selective barrier: intimal collagen is exposed and digested; medial collagen is protected. In advanced lesions where the lamina is fragmented, this stage is shortened or omitted, guided by the effluent hydroxyproline signal. The effluent signal for this stage is hydroxyproline concentration, measured by colorimetric assay. A limitation of effluent-based monitoring is that it provides a bulk measurement averaged over the entire treatment zone. If the internal elastic lamina is intact over most of the segment but fragmented at a single point, medial collagen digestion at that point may be diluted below the detection threshold in the total effluent volume. Conservative dosing and exposure times for collagenase are therefore necessary. Collagenase from *C. histolyticum *is immunogenic; Xiaflex carries a hypersensitivity warning. In the isolated chamber, the enzyme does not reach systemic circulation during treatment. However, residual enzyme adsorbed on the treated vessel wall will contact blood upon balloon deflation. The final saline flush must reduce residual collagenase below the immunogenic threshold, a threshold that decreases with repeated exposure due to sensitization. Quantifying residual enzyme after flushing and monitoring for anti-collagenase antibodies between sessions are necessary safety measures. In patients who develop sensitization, subsequent sessions would omit the collagenase stage and rely on EDTA and surfactant alone, accepting incomplete fibrous cap removal.

Stage 4: Optional Acid Soak

Buffered saline at pH 4.5 dissolves residual hydroxyapatite microcrystals embedded in the fibrous matrix newly exposed by collagenase. Exposure is kept brief. Collagen and elastin are acid-stable well below this pH.

Stage 5: Saline Flush

A copious bidirectional saline flush is then performed until the effluent is indistinguishable from input saline across all monitored parameters. Before blood is reintroduced, a final heparin-bonded saline rinse coats the denuded vessel surface with an antithrombotic layer. This step is critical: the treated surface presents exposed subendothelial collagen and tissue factor, a maximally thrombogenic environment, more so than standard angioplasty because the exposure area is larger and the surface has been enzymatically treated. Once the chamber is ready, blood from the bypass circuit is admitted to refill the treated segment. Balloons are then deflated, restoring native arterial flow. The bypass circuit is discontinued. Catheters are removed. Hemostasis is achieved by compression or by deployment of a vascular closure device.

Summary of staged dissolution

Table 1 summarizes the agents, their plaque targets, the self-stopping mechanism at the internal elastic lamina, and the corresponding effluent diagnostic for each stage.

**Table 1:** Staged chemical dissolution protocol Each stage targets a specific plaque component, self-stops at the IEL through a chemistry-specific mechanism, and produces an effluent signal that serves as both endpoint indicator and quantitative plaque characterization. Stages 1-3 are cycled iteratively until all effluent channels return to baseline simultaneously. EDTA: ethylenediaminetetraacetic acid; IEL: internal elastic lamina; HP-β-CD: hydroxypropyl-β-cyclodextrin

Stage	Agent	Plaque target	Self-stopping basis	Effluent diagnostic
1	EDTA, 50-100 mM	Hydroxyapatite (calcified deposits)	Absent from IEL	Ca2+ (ion-selective electrode)
2	Poloxamer 188 (1-5%) or HP-β-CD (10-50 mM)	Lipid-rich necrotic core	Absent from IEL	Turbidity, lipid colorimetry
3	Collagenase (*C. histolyticum*)	Fibrous cap collagen (types I and III)	Does not cleave elastin (IEL)	Hydroxyproline
4	Buffered saline, pH 4.5	Residual hydroxyapatite microcrystals	Elastin acid-stable	Ca2+
5	Saline (heparin-bonded)	Flush; antithrombotic coating	N/A (rinse stage)	All channels return to baseline

Self-stopping at the internal elastic lamina

Every chemical agent in the protocol is independently self-stopping at the internal elastic lamina, the boundary between the plaque-bearing intima and the structural media of the vessel wall. EDTA targets hydroxyapatite, which is absent from the normal lamina. Surfactant targets accumulated lipid, absent from the elastin lamina. Collagenase cleaves collagen but does not cleave the elastin that constitutes the lamina. The optional acid soak dissolves hydroxyapatite but not elastin. Three chemically distinct agents arrive at the same tissue boundary, each from a different direction, and each stops for a different biochemical reason. This convergence is not an engineering constraint imposed on the system: it is a property of the agents and the tissue.

In advanced lesions where the lamina is fragmented, the effluent signals serve as the primary depth control: rising hydroxyproline from collagenase indicates medial collagen exposure and signals termination of that stage.

A related constraint applies to heavily diseased segments where atherosclerotic plaque has become load-bearing, where the plaque effectively is the vessel wall. In such segments, complete plaque removal would leave a structurally inadequate wall unable to withstand arterial pressure. The protocol accommodates this through its iterative design: each session removes a partial thickness of plaque, and the wall undergoes adaptive remodeling during the inter-session interval before additional material is removed. The expected remodeling mechanism is neointimal hyperplasia, smooth muscle cell proliferation and extracellular matrix deposition in response to the partial intimal disruption [13]. This process, usually considered pathological in the context of post-angioplasty restenosis, here serves a structural function: it thickens and strengthens the partially cleared wall before the next session. Subsequent sessions then remove both residual original plaque and a fraction of the new neointimal tissue, progressively replacing diseased wall with remodeled tissue over multiple cycles. Complete single-session removal is neither attempted nor desirable in advanced disease.

Endothelial disruption and regeneration

The endothelium overlying atherosclerotic plaque will be disrupted during treatment. This is an inevitable consequence of dissolving the substrate on which the endothelial cells sit. Endothelial cells are, however, among the fastest-regenerating cell populations in the body, with re-endothelialization of denuded segments occurring within days to weeks [14]. The media remains structurally intact because none of the agents attack its components at the concentrations and exposure times used.

Temporary endothelial disruption is not only tolerable but functionally necessary for the iterative protocol: healing endothelium with transiently upregulated adhesion molecules is precisely what makes cleaned sites preferential traps for circulating lipid mobilized from inaccessible sites. Complete re-endothelialization between sessions restores vascular function; the next session re-exposes the subendothelial surface. The cycle is biologically natural: it recapitulates, under controlled conditions, the same endothelial injury-and-repair sequence that occurs after every conventional balloon angioplasty.

Treatment as diagnosis

The staged protocol generates a quantitative, component-resolved plaque characterization as a byproduct of treatment. The EDTA stage effluent calcium concentration quantifies calcification burden. The surfactant stage effluent turbidity and lipid content quantify necrotic core volume. The collagenase stage effluent hydroxyproline quantifies fibrous cap mass. Per-direction analysis of all markers provides spatial distribution.

No existing imaging modality, such as intravascular ultrasound, optical coherence tomography, or computed tomography angiography, provides simultaneous quantitative resolution of calcified, lipid, and fibrous components with spatial directionality. This diagnostic output requires no additional hardware; it falls out of the treatment protocol.

Iterative protocol: draining inaccessible sites

After chemical cleaning, the treated wall presents exposed subendothelial matrix with healing but not yet recovered endothelium. This surface is biochemically receptive to circulating lipoprotein deposition. Healing endothelium transiently upregulates adhesion molecules as part of the repair process, increasing local lipoprotein retention. Meanwhile, pharmacological mobilization drives lipid and calcium from inaccessible deposits (cerebral microvasculature, coronary microvessels, retinal vasculature, deep visceral beds) into systemic circulation.

The redeposition principle is presented here as a hypothesis rather than a demonstrated mechanism: cleaned accessible sites are predicted to be preferred for redeposition over fully loaded inaccessible sites for three reasons, namely, available binding sites on exposed matrix, upregulated adhesion molecules on healing endothelium, and large-vessel hemodynamic geometry that favors deposition at the same branch point sites where plaque originally accumulated [6]. The liver would be expected to clear a fraction of mobilized lipid via the low-density lipoprotein (LDL) receptor pathway; the remainder is hypothesized to redeposit at cleaned accessible sites, where it would be available for extraction in the next session. The quantitative partitioning between these fates is an empirical question that testable prediction 5 is designed to address. The protocol provides direct mechanical clinical benefit at the accessible large vessels independently of whether the redeposition-and-extraction cycle holds quantitatively; the iterative drainage of microvascular sites is the additional benefit if it does.

The session sequence proceeds as follows.

Session 1

Chemically clean all accessible large and medium vessels at their branch points and stenosis sites. Each segment is cleaned gently, using conservative endpoints and minimal wall exposure. Roughly 60-70% of total body plaque burden by mass resides in these accessible vessels; a single comprehensive session targets a 30-50% removal of this accessible fraction conservatively, corresponding to 18-35% of total body plaque burden in the first session. Aggressive single-session removal would risk wall injury and is not pursued; cumulative reduction is the goal of the iterative protocol. The expected duration of a single session ranges from 30 to 90 minutes per bifurcation segment, with a complete multi-segment session running 2-4 hours overall, comparable to a multi-vessel percutaneous coronary intervention. Per-segment durations are constrained by the ischemic tolerance of territories supplied by minor branches within the isolated segment: peripheral and renal applications fall within standard ischemic tolerance windows, while cerebrovascular and mesenteric applications would require shortened sessions or staged approaches.

Between cleaning sessions, an interval of weeks to months allows pharmacological mobilization to drive material from inaccessible sites into the systemic circulation. Mobilized material redeposits at cleaned sites. The duration of this interval is guided by serial lipid panels and inflammatory markers. High-sensitivity C-reactive protein (hsCRP) and interleukin-6 (IL-6) measure systemic inflammation predictive of cardiovascular events. Lipoprotein-associated phospholipase A2 (Lp-PLA2) is a vascular-specific marker indicating plaque instability and inflammation. Interval ends when biomarkers plateau.

Session 2

Re-clean accessible sites. Extracted material includes residual plaque and newly deposited material from inaccessible sites. Total extracted burden exceeds session 1 residual, confirming net drainage. Subsequent sessions are repeated until the effluent shows negligible dissolved material, indicating depletion of the systemic plaque reservoir.

The protocol converges because each session removes more material than the subsequent interval replaces from a diminishing inaccessible reservoir and because the systemic inflammatory environment that drives new deposition collapses with each session. Large atheromata are the dominant source of IL-6, TNF-α, oxidized LDL, and monocyte chemoattractant protein-1. Removing them reduces circulating inflammatory mediators and the rate of new deposition throughout the vascular tree. The remaining small-vessel disease loses both its growth stimulus and its lipid supply. The final residual fraction ceases to grow and becomes susceptible to pharmacological clearance alone.

Convergence depends on a sufficient fraction of pharmacologically mobilized lipid redepositing at cleaned accessible sites rather than being cleared hepatically or depositing at uncleaned sites. The quantitative partitioning among these fates is an empirical question. The protocol still provides clinical benefit even if hepatic clearance dominates, since each mechanical session removes the burden directly.

Atherosclerosis is predominantly a disease of large and medium arteries; the majority of total plaque mass resides in vessels that are accessible by the catheter-based approach described here. The inaccessible microvascular compartment carries a smaller but non-trivial burden. Direct cleaning addresses the accessible fraction over the first sessions. The iterative redeposition-and-extraction cycle progressively drains the microvascular remainder into the accessible domain, where it is extracted at subsequent sessions. Residual burden that neither redeposits nor responds to the inflammatory collapse remains a target for conventional pharmacological clearance, now operating against a dramatically reduced reservoir. Quantifying the mass fractions associated with each compartment, and the redistribution efficiency of the iterative cycle, is among the primary objectives of the proposed animal studies. The clinical consequences of restoring normal lumen geometry and perfusion across the major vascular beds are established and motivate the experimental validation proposed here.

Pharmacological mobilization protocol

Between sessions, aggressive pharmacological mobilization drives lipid and calcium from inaccessible deposits into systemic circulation, where they are either cleared hepatically or redeposited at cleaned accessible sites for extraction at the next session.

Lipid mobilization uses PCSK9 inhibitors (evolocumab or alirocumab). Proprotein convertase subtilisin/kexin type 9 (PCSK9) is a hepatic protein that regulates blood cholesterol by controlling the lifespan of hepatic LDL receptors; PCSK9 inhibitors prevent receptor degradation, maximally upregulating hepatic LDL clearance. LDL-C target under 30 mg/dL. The Global Assessment of Plaque Regression with a PCSK9 Antibody as Measured by Intravascular Ultrasound (GLAGOV) trial, a phase 3 study using evolocumab, demonstrated that PCSK9 inhibition achieves measurable plaque regression of the lipid-rich component [15] but not of calcified plaque, which the present system addresses directly. High-intensity statin (rosuvastatin 40 mg or atorvastatin 80 mg) provides complementary LDL reduction and pleiotropic anti-inflammatory effects.

Calcium mobilization uses high-dose menaquinone-7 to saturate matrix Gla protein carboxylation, mobilizing established vascular calcification and directing calcium to bone via activated osteocalcin. The target dose (1 g per day) represents an approximately 3,000-fold extrapolation from studied clinical doses (up to 360 micrograms per day) and requires formal dose-finding with monitoring of coagulation parameters, hepatic function, and serum calcium before clinical use. This regimen is contraindicated in patients taking vitamin K antagonists. Cholecalciferol (10,000 IU per day) drives osteocalcin production and skeletal calcium demand, with periodic 25-hydroxyvitamin D monitoring.

Serial lipid panels and inflammatory markers (hsCRP, IL-6, Lp-PLA2) guide the inter-session interval. The interval ends when biomarkers plateau, indicating redistribution equilibrium.

Testable predictions

The hypothesis generates six experimentally testable predictions.

First, EDTA at 50-100 mM in a balloon-isolated, blood-free porcine arterial segment produces effluent Ca²⁺ concentration exceeding input saline by at least 1 mM within 30 minutes, as quantified by ion-selective electrode, with pre- and post-treatment intravascular ultrasound confirming plaque volume reduction.

Second, poloxamer 188 at 1-5% weight by volume emulsifies lipid-rich plaque components in the same model, as quantified by effluent turbidity and lipid assay.

Third, collagenase at 0.1-0.5 mg/mL digests fibrous cap selectively without penetrating an intact internal elastic lamina, as quantified by effluent hydroxyproline and post-treatment histology.

Fourth, the bidirectional flow protocol achieves more complete plaque removal than unidirectional flow in matched segments, as quantified by post-treatment intravascular ultrasound.

Fifth, cleaned arterial segments in a hyperlipidemic animal model accumulate more circulating lipid than unclean contralateral controls over a 4-8-week interval, confirming the redeposition principle. This prediction tests the central hypothesis of the iterative protocol: that healing endothelium with upregulated adhesion molecules creates a preferential trap for mobilized lipid. Quantification is performed by post-treatment intravascular ultrasound assessment of lipid burden, supported by histological analysis of cleaned versus control segments at study endpoint. A statistically significant difference (cleaned > control by at least 50% in lipid burden; p<0.05 across n≥6 paired comparisons) would validate the redeposition mechanism.

Sixth, serial sessions in the same model show decreasing plaque burden per session, confirming convergence. This prediction tests the protocol's iterative depletion of the systemic plaque reservoir. Quantification is performed by tracking total effluent mass per session across at least three sequential sessions in the same animal. The expected pattern is a monotonic decrease in extracted material from session to session. A consistent downward trend (each session extracting less than the prior, with cumulative extraction approaching an asymptote) would validate convergence and distinguish the protocol from a single-session intervention.

Each prediction is testable in a porcine atherosclerosis model using existing equipment and established protocols. No novel fabrication is required. The first four predictions can be tested in a single series of acute experiments.

## Discussion

Procedure logistics

Treatment of a single bifurcation segment requires an estimated 30-90 minutes depending on plaque burden and the number of chemical cycling passes needed. A full first session addressing all major accessible bifurcations would be performed sequentially over multiple hours under conscious sedation with local anesthesia at each access site, comparable to a multi-vessel percutaneous coronary intervention, which routinely runs 2-4 hours. General anesthesia is not required.

Regulatory path

Every system component has regulatory precedent: percutaneous balloon catheterization, extracorporeal bypass circuits, EDTA infusion [8], poloxamer 188 infusion [11], injectable collagenase [12], and effluent monitoring instrumentation. The novel element is the combination and the iterative protocol. The path to human use begins with a physician-initiated clinical trial under institutional review board approval, following successful demonstration in the porcine atherosclerosis model. Outcome data from early trials would support regulatory filing for broader access. The translational pathway parallels other complex cardiovascular and metabolic protocols that combine multiple technologies and require iterative refinement through preclinical porcine studies before human application [16].

Limitations

This is a concept paper with no experimental validation. The following limitations and uncertainties are acknowledged. (a) EDTA dissolution kinetics for consolidated calcification may be slower than the treatment window permits; the cycling protocol is expected to accelerate effective rates, but this requires empirical confirmation. (b) Collagenase depth control via bulk effluent hydroxyproline has limited spatial resolution and may not detect focal breaches of the internal elastic lamina. (c) The quantitative partitioning of pharmacologically mobilized lipid among hepatic clearance, redeposition at cleaned sites, and redeposition at uncleaned sites is unknown. (d) The menaquinone-7 dose (1 g per day) represents an approximately 3,000-fold extrapolation from studied clinical doses and requires formal dose-finding. (e) Heavily calcified, eccentric vessels may not seal adequately with compliant balloons and may be unsuitable for this protocol. (f) The long-term vessel wall response to iterative chemical treatment (multiple cycles of partial plaque removal, neointimal formation, and re-treatment) is unknown and can only be characterized in longitudinal animal studies. (g) Collagenase immunogenicity may preclude its use in later sessions if patients develop sensitization. (h) Several mechanistic arguments rely on extrapolation from related but non-identical contexts: dissolution rate data from in vitro hydroxyapatite studies under stirring conditions [9], surfactant safety from intravenous use in different chemical environments [11], and inflammatory-mediator dynamics from cohort studies of natural disease progression. These extrapolations are necessary at the concept stage but require direct validation in the proposed experimental framework. (i) This work does not include a quantitative statistical framework or computational model of the iterative protocol's convergence dynamics. Such a model, tracking plaque mass partition between accessible and inaccessible compartments across sessions, is a prerequisite for clinical trial design and is left to follow-on work informed by the porcine experiments. (j) The iterative redeposition-and-extraction hypothesis is the most speculative element of the protocol. Its empirical confirmation in a hyperlipidemic animal model is testable prediction 5. The protocol's clinical utility at the accessible vessel level does not depend on this hypothesis; the hypothesis governs only the protocol's reach into the microvascular compartment.

## Conclusions

Topological isolation of a vascular segment converts interventional atherectomy from a problem of debris management in flowing blood into a problem of chemical dissolution in a sealed saline chamber. Within this chamber, concentrated chelators, surfactants, and enzymes that could not be deployed in systemic circulation can be applied at concentrations sufficient to dissolve the major components of atherosclerotic plaque at their respective chemical vulnerabilities. Cycling the agents iteratively is proposed to penetrate layered atheromata. Bidirectional flow eliminates geometric shadow zones. Real-time effluent chemistry provides treatment endpoint and quantitative plaque composition. The iterative session-and-interval protocol is hypothesized to use healing-induced redeposition at cleaned sites to progressively drain plaque from inaccessible microvascular beds; this hypothesis requires empirical validation. Every system component has an existing regulatory precedent. Six testable predictions provide a path to validation in a porcine atherosclerosis model.
